# Complete Traumatic Luxation of the Eyeball

**DOI:** 10.18502/jovr.v16i4.9760

**Published:** 2021-10-25

**Authors:** Manpreet Singh, Amandeep Singh Jassi, Manpreet Kaur, Pankaj Gupta

**Affiliations:** ^1^Department of Ophthalmology, Advanced Eye Centre, Post Graduate Institute of Medical Education and Research, Chandigarh, India; ^2^Department of Radiodiagnosis, Accuscan Diagnostic Centre, Mohali, Punjab, India

**Keywords:** Eyeball Displacement, Globe Luxation, Globe Subluxation, Ocular Trauma, Ophthalmic Trauma

## Abstract

**Purpose:**

To report the computed tomography features of a case with complete luxation of the globe after a road traffic accident.

**Case Report:**

A 35-year-old male presented with pain, loss of vision, and bleeding from the left eye 48 hr after a road traffic accident. The ophthalmic examination of the left upper and lower eyelids showed edema with subcutaneous hematoma, crepitus, and complete blepharoptosis. On retracting the eyelids, the left eyeball was not visible and the patient was not able to perceive light. The left temporal region appeared filled with a soft, palpable globular structure situated beneath the temporalis muscle. A non-contrast computed tomography (NCCT) of the head and orbits showed a comminuted and displaced fracture of the floor, medial, and lateral orbital walls in addition to a displaced tripod fracture of the left zygomatic bone. The intact left eyeball was seen below the temporalis muscle without any optic nerve or extraocular muscle attachment. The virtual reality reconstruction highlighted a contributory supero-temporal defect in the bony orbit, which appeared large enough to accommodate the intact eyeball.

**Conclusion:**

The computed tomography of the orbits provided a detailed location of the luxated eyeball and provided guidance in further management of the case.

##  INTRODUCTION 

A complete luxation of the eyeball is a rare and devastating ophthalmic trauma, which is occasionally accompanied by traumatic avulsion of the optic nerve and extraocular muscles.^[1,2]^ Morris et al explained this mechanism in three categories: direct impact, wedge effect, and lever-fulcrum action.^[2]^ Orbital computed tomography (CT) and magnetic resonance imaging revealed details of fractures, the location of the eyeball, optic nerve, and extraocular muscle attachments.^[1,2]^

**Figure 1:**
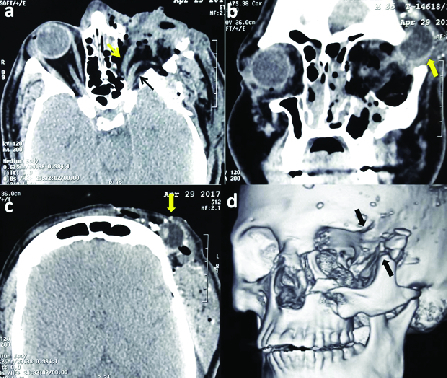
Computed tomography images of the orbit, brain, and 3D face reconstruction. (a) Axial scan illustrates left orbital fractures, pneumo-orbit, lost eyeball with transected medial rectus (yellow arrow), and optic nerve (black arrow). (b) The coronal section shows a luxated eyeball (yellow arrow) through the fracture space, pneumo-orbit, and left maxillary hemosinus. (c) The superior axial section shows the luxated eyeball beneath the anterior fibers of left temporalis muscle with air pockets and multiple small bone fragments. (d) The virtual reality image illustrates the left displaced tripod fracture of the zygomatic bone and a contributory wide bony space created (two black arrows).

##  CASE REPORT

A 35-year-old male presented to ophthalmology emergency suffering with pain, loss of vision, and bleeding from his left eye for 48 hr after experiencing a road traffic accident whilst under the influence of alcohol. On presentation, the patient was calm, conscious, co-operative, and oriented to time, place, and person. His neurological examination was within normal limits. On ophthalmic examination, the right eye appeared normal with a visual acuity of 6/6. The left upper and lower eyelids were edematous with subcutaneous hematoma and complete blepharoptosis. Crepitus was felt on the palpating eyelids. On retracting the eyelids with a Desmarres retractor, the left eyeball was not visible, and the patient was not able to perceive light. The left temporal region appeared filled with a soft, palpable globular structure situated beneath the temporalis muscle.

A non-contrast computed tomography (NCCT) of the head and orbits was requested. The NCCT axial images revealed a comminuted and displaced fracture of the medial and lateral orbital walls [Figure 1a]. The coronal section also revealed a displaced tripod fracture of the left zygomatic bone and orbital floor [Figure 1b]. The intact left eyeball was seen below the temporalis muscle without any optic nerve or extraocular muscle attachment [Figure 1c]. The virtual reality reconstruction highlighted a contributory supero-temporal defect in the bony orbit, which appeared large enough to accommodate the intact eyeball [Figure 1d].

After receiving informed consent, the removal of the eyeball from the sub-temporalis space was performed. Intraoperatively, the eyeball was found to be hypotonus with a surrounding purulent foul-smelling discharge. The outer scleral surface was necrosed and the cornea was opaque. No optic nerve or muscle stump was identified. The specimen was sent for histopathological and microbiological examination. An open reduction internal fixation surgery using mini-plates was performed for the tripod fracture repair and reconstruction of the bony orbital rim. In order to achieve the best possible cosmetic rehabilitation, a second-stage orbital volume restoration using a dense porous polyethylene spherical implant was planned, to be followed by a customized ocular prosthesis.

##  DISCUSSION 

A subluxated eyeball is routinely reposited back in the orbit, surgically or with Tse's maneuver (for spontaneous subluxation).^[3]^ However, complete luxation of the eyeball accompanied with detachment of all recti and optic nerves justifies its removal, given its lost vitality and the presence of obvious future implications. The seven branches of muscular arteries, ciliary arteries (long – 2, short – 6 to 12) and the retinal artery, which contributes to the majority of the ocular blood supply, gets detached from the luxated eyeball. The loss of arterial blood supply initiates necrosis and enhances tissue infection. In our detailed published text entitled “subluxated globes”, we have comprehensively covered the etiopathogenesis, clinical features, and management of this disastrous condition.^[4]^ We pointed out that orbital imaging provides vital information about the position of subluxated or luxated eyeballs, and the extent of surrounding tissue damage and orbital bone fractures.^[4]^ The joint management of luxated eyeball cases by ophthalmologists, maxillo-facial surgeons, and neurosurgeons provides a holistic approach in achieving the best possible cosmetic and functional outcomes.

In summary, the CT used to assess this particular case of luxation provided valuable information in determining the location of the eyeball, the detachment of arteries, the presence of orbital fractures and ocular related injuries.

##  Financial Support and Sponsorship

Nil.

##  Conflicts of Interest

There is no conflict of interest.
